# Medical ethics and the trolley Problem

**Published:** 2019-03-17

**Authors:** Gabriel Andrade

**Affiliations:** *Assistant Professor, Department of Ethics and Behavioral Science, School of Medicine, St. Matthew’s University, Cayman Islands.*

**Keywords:** Trolley problem, Medical ethics, Non-maleficence, Abortion, Euthanasia

## Abstract

The so-called Trolley Problem was first discussed by Philippa Foot in 1967 as a way to test moral intuitions regarding the doctrine of double effect, Kantian principles and utilitarianism. Ever since, a great number of philosophers and psychologists have come up with alternative scenarios to further test intuitions and the relevance of conventional moral doctrines. Given that physicians routinely face moral decisions regarding life and death, the Trolley Problem should be considered of great importance in medical ethics. In this article, five “classic” trolley scenarios are discussed: the driver diverting the trolley, a bystander pulling a lever to divert the trolley, a fat man being thrown from a bridge to stop the trolley, a bystander pulling a lever to divert a trolley so that a fat man may be run over, and a bystander pulling a lever so that a fat man falls off from a bridge to stop the trolley. As these scenarios are discussed, relevant moral differences amongst them are addressed, and some of the applications in medical ethics are discussed. The article concludes that Trolley scenarios are not the ultimate criterion to make ethical decisions in difficult ethical challenges in medicine cases but they do serve as an initial intuitive guide.

## Introduction

***The Primacy of Non-Maleficence in Medical Ethics***

With some oversimplification, it could be argued that medical ethics is about balancing the four main principles long recognized as central in medical practice throughout the ages: autonomy, beneficence, non-maleficence, and justice (1). It is commonly agreed that, ever since the beginnings of ethical reflection, non-maleficence has been the most important of all principles, and should be given priority when in conflict with others (2).

Although Hippocrates did not explicitly mention the phrase “first do no harm” in his Oath (the original version actually states, “Abstain from doing harm”), it is enshrined in the common medical understanding of ethics (3). And indeed, this principle prevails above others.

Take, for example, the case of a person suffering from minor pain, who asks her doctor for a massive administration of morphine. When considering the principle of autonomy, it would seem that the right thing to do is to, indeed, comply with the patient’s request. But, as much as morphine is a dangerous substance with great abuse potential, the physician should be aware that its administration will ultimately cause great harm. In this case, autonomy would be at odds with non-maleficence, and the doctor must privilege the latter over the former. First, do no harm. Although the patient may desire a specific procedure, the physician is required to think about whether or not that particular procedure will be harmful for the patient. If in conscience the doctor does believe that the procedure will be harmful, then it should not be prescribed, even if the patient asks for it.

Non-maleficence may also be at odds with beneficence. Most doctors have the legitimate desire to do good, but as the popular saying goes, the road to hell may sometimes be paved with good intentions. Some procedures may appear to be good in the short term, but they could have very prejudicial consequences in the long term (or perhaps even in the short term). Again, the prime principle in medical ethics is first doing no harm. If, by trying to address a health problem out of a concern with beneficence, the doctor puts the patient in an even worse condition, then that procedure should not be done. That is why, amongst other things, when it comes to new biotechnologies, most ethicists prescribe a cautionary principle (4). For the most part, we do not have full knowledge of how some of the newer biotechnologies work, and it is therefore better to suspend the administration of those biotechnologies until further knowledge about their workings is gathered. Even if those biotechnologies offer good solutions to particular health problems, they may in fact cause even greater harm.

Justice may also clash with non-maleficence, and again, the latter should take precedence. The just allocation of resources in health care may sometimes imply procedures that will ultimately do more harm to patients. Consider, for example, a famous case put forth by philosopher Philippa Foot and ever since widely popularized: A surgeon has five patients who are waiting for organ transplants. The patients will die if they do not receive the organs, but the organs are not available at the time. The whole prospect changes, however, when a young traveler comes to town and goes in for a routine checkup. The doctor is performing the checkup when he realizes that the traveler’s organs are healthy and incidentally compatible with his dying patients. The young man is the perfect donor, and no one would associate him with the surgeon if he were to disappear (5). The dilemma here is, should the doctor remove the organs from the healthy man in order to distribute them to the dying patients? Of-course not. Although it may be a more efficient and even just allocation of resources, it would still be a moral monstrosity to authorize such a transplant. The reasoning here is that non-maleficence takes precedence over the other ethical principles.

It is safe to argue, then, in favor of the primacy of non-maleficence, but medical ethicists seldom ask why that should be the case. What authorizes first doing no harm? It seems that much of this reasoning relies on strong intuitive appeals. Nevertheless, these intuitive appeals face an important critique, as there may be some additional intuitions illustrated by other cases, in which it does not seem to be so clear that non-maleficence should take primacy.

## Discussion

***1.      The First Trolley Case***

Philosophers conventionally call the “Trolley Problem” a series of bizarre questions and dilemmas that derive from some particular situations that elicit moral responses. They make reference to a trolley because, in the variants, the cases are about trolleys going down the tracks, and on their course, they run down persons who are helplessly tied to the tracks. The purpose of these cases is to test intuitions, so as to decide what actions are morally correct. These intuitions can be extended to cases (mostly medical, although by no means exclusively so, for they may also have military applications) that are structurally similar, and on that basis, we may decide what the proper course of action is.

The first trolley scenario was proposed by Philippa Foot (6), and is about a trolley that is going down the tracks, and is set on course to run down five people who are tied to the tracks. The driver of the trolley has the option to divert the trolley onto another track in which only one person is tied. Foot wondered whether or not the driver should divert the trolley.

Foot answered that, indeed, the driver should divert the trolley. A simple calculation shows why this is so. If the driver keeps the trolley on its tracks, five people will be run over and die. If, by contrast, the driver diverts the trolley, only one person will die. It seems ethically acceptable to kill one person in order to save five.

Nonetheless, Foot herself warned that it is not always ethically fine to kill one person to save five. Consider the case of the transplant mentioned above: a surgeon thinks about a healthy person who shows up at a hospital, and five terminally ill patients who can get cured with that person’s organs. Should that person be killed so that the other five survive?

Surveys show that, overwhelmingly, respondents disapprove of such a transplant (7). Foot also disapproved, yet she wondered why in the case of the trolley it is morally acceptable to kill one in order to save five, whereas in the case of the transplant it is not morally acceptable to kill one in order to save five.

Her answer relied on a distinction between negative duties and positive duties. We have duties *not *to do certain things, and duties to do certain things (i.e., negative and positive, respectively). In Foot’s estimation, negative duties are more important than positive duties, and if they ever come into conflict, negative duties should be given priority.

In the case of the transplant, there is indeed the positive duty to help the five patients. However, there is the even greater negative duty of *not *harming the healthy person. Although Foot does not address this issue, we may even ask whether the healthy person’s consent would justify killing him in order to transplant his organs to save the five. The standard answer in medical ethics would be that, even in that case, it would be unethical for a doctor to carry on such a procedure. Again, non-maleficence overrides autonomy.

In the case of the transplant, the dilemma is between killing one and letting five die. Foot clearly argues that there is no moral justification to kill the one person, as killing is a greater offence than letting die, even if five are left to die, and only one is killed. The negative duty towards the one is greater than the positive duty towards the five.

Yet, how is this different from the driver who diverts the trolley, thus killing one in order to save five? Foot argues that, in this case, the dilemma is different. It is no longer a dilemma between killing one and letting five die, but rather, between killing one and killing five. By default, the driver’s original action (setting the trolley in movement) will end up in killing five people. He may choose to take another action, and thus kill one. In both scenarios, his action will ultimately kill someone. If that is the case, then it is better to kill one than to kill five. The driver has the duty not to kill anybody. But, given that his actions will ultimately kill someone, the lesser evil is to kill as few people as possible. Therefore, he is morally required to switch the trolley onto another track.

This first variant of the Trolley Problem supports the primacy of non-maleficence in medical ethics. The five patients may die as a result of the transplant not taking place, but the surgeon is not ethically at fault since he has done no harm, and that is a doctor’s most important duty. In order to save the five, he would have had to kill the one person. The surgeon wisely refuses to engage in such a procedure in deference to non-maleficence.

The driver of the trolley, by contrast, does have the moral obligation to kill one in order to save five, because those five will die as a result of his own initial action. As opposed to the doctor, the driver is not in a position to claim that his duty is to first do no harm. This is because the driver already has done some harm by setting the trolley on course to kill five people. His moral duty is to take additional action to minimize his initial harm. Killing one is not better than letting five die, but killing one is indeed better than killing five.

Foot’s reasoning (and, as a corollary, the primacy of non-maleficence in medical ethics) relies on the assumption that there is a significant difference between omissions and actions, and this corresponds with negative duties vs. positive duties. Yet, this has been challenged by some philosophers, notably James Rachels (8). Foot believes there is an important ethical difference between killing and letting die. Rachels, by contrast, believes the difference is not significant.

Consider the following case as an instance: A woman desires her uncle to die, and administers poison in his coffee. Another woman also wants him dead, and is about to give him poison, but then she notices that he drinks poison from another source. She then observes him dying, and withholds the antidote in her pocket.

Rachels argues that, in this case, neither of the women is worse than the other, and intuitively, he seems to be right. According to Rachels, this indicates when it comes to killing and letting die, there is no significant difference. It also proves that there is no major difference between negative and positive duties. Rachels thus subscribes the Equivalence Thesis regarding killing and letting die.

If Rachels is right, then his claim has big implications on medical ethics, and the primacy of non-maleficence can be put into question. Rachels himself has been a defender of euthanasia. Medical ethicists conventionally differentiate between passive euthanasia and active euthanasia. Passive euthanasia proceeds by letting patients die (for example by withholding treatment or disconnecting artificial ventilators), whereas in active euthanasia death is induced through additional procedures, such as administering specific substances. 

Medical ethicists typically allow for passive euthanasia if the patient consents, but condemn active euthanasia even if the patient consents (9). For the most part, legislations also support this moral stand. In no country is it illegal to withhold treatment if it is the patient’s wish, but in the overwhelming majority of countries it is illegal to actively induce death, even if the patient asks for it. The moral rationale is that there is a difference between killing and letting die, and therefore, this supports the primacy of non-maleficence.

Yet, if Rachels is right and his example is intuitive and powerful enough, the difference between killing and letting die collapses, and as a result, non-maleficence may not be as primal as traditionally thought. Sometimes, it may be morally acceptable to actively do harm, for instance by killing someone in order to stop that person from suffering.

Although Rachels’ hypothetical scenario is intuitive, there are plenty of other scenarios that lead our intuitions towards the original idea that killing and letting die are two very different things. There is a fundamental difference between murdering someone and letting hundreds of unfed children die in some Third World country due to indifference. We may have the moral obligation to care for those children, but it seems that that neglect will never be morally equivalent to murder.

***2.      The Second Trolley Case***

Intuitions, as laid out in Foot’s arguments, seem to support the primacy of non-maleficence, which justifies not killing a healthy person to distribute his organs to five sick patients. Even when harm is already done, there is justification to seek the lesser harm, as in the case of diverting the trolley to kill one instead of five.

In order to test new intuitions, philosophers have further come up with additional trolley cases. As it turns out, in some cases, it seems like causing harm is the right thing to do. Consider, for example, a variant of the Trolley Problem devised by Judith Jarvis Thomson (10).

In this scenario, very much as in the first one, a trolley is going down its path and it will run over five people. There is the option of diverting the trolley onto another track in which one person is tied. However, the difference in this case is that it is not up to the driver, but rather to someone standing by, to switch the trolley by pulling a lever. Should the bystander pull the lever?

The fact that the decision now has to be made by a bystander and not the driver is of great importance, as the bystander faces a different dilemma. In Foot’s analysis, the driver must decide whether he should kill five people or one, and that is why it seems morally acceptable for him to divert the trolley. The bystander, however, was not responsible for setting the trolley on its original course in the first place, and if the trolley runs over five people by going down its original path, it will not be his responsibility. If, instead, he diverts the trolley on the track to kill the one person, then it will be his responsibility. Thus, the bystander’s dilemma is not killing one versus killing five, but rather, killing one versus letting five die.

We have already established that, putting Rachels’ objections aside, there seems to be a significant difference between killing and letting die. That is why killing one is worse than letting five die. This explains well the moral intuitions most people have when it comes to a healthy person being killed so that his organs are distributed amongst five patients.

Yet, surveys consistently show that in the case of the bystander who has the option of pulling the lever to divert the trolley and kill one person in order to save five, the overwhelming majority of respondents have the intuition that the bystander would be morally obligated to pull the lever (11). Somehow in this case, letting five die is worse than killing one.

The intuition in favor of the primacy of non-maleficence, then, does not seem as strong as it originally appeared. Perhaps in some cases, the rule of first doing no harm can be relaxed. The bystander would unmistakably be causing harm by pulling the lever and killing the one person on the diverted track, but he would most likely be lauded.

Yet, even if the principle of non-maleficence may be somewhat relaxed, there is still a need to be precise about when such relaxation can take place. In part, this can be done by trying to find a difference between the case of the surgeon who intends to kill a person so that five patients get his organs, and the case of a bystander who pulls the lever to divert a trolley to kill one person instead of letting five die. In both cases, the dilemma is between killing one and letting five die, yet intuitively, the morally right thing to do is very different.

The standard philosophical answer (the one tentatively provided by Thomson herself) is that, although in both cases the dilemma is between killing one and letting five die, there is a crucial difference. In the case of the surgeon seeking to kill a person to distribute his organs to five patients, that person is being used as a means to an end. In turn, in the case of the bystander who pulls the lever to divert the train to kill one person, that person would die as an unfortunate side effect of the bystander’s decision, but would not be used as a means to an end.

If, somehow, the person in the hospital could escape, the surgeon’s plan to save the five patients would be shattered. In that sense, the person in the hospital becomes a means to save the five patients. If instead, somehow the one person in the track could escape, that would not shatter the bystander’s plan to save the five that are tied to the other track. In that sense, the one person in the track does not become a means to save the other five.

This distinction relies on Kant’s moral philosophy (12). Kant famously argued that part of the moral imperative is never to treat other people as means to ends, even if those ends are praiseworthy. Kant’s philosophy is emblematically deontological, as opposed to utilitarian. Deontological ethics prescribes that moral agents do the right thing on the basis of duty, regardless of the consequences, or as the poetic phrase goes, “even if the heavens fall” (13). Utilitarian ethics, instead, allows for more accommodation, as long as the end results bring about a higher quantity of good; utilitarian ethics are consequentialist, in the sense that the worth of an action is not in its intrinsic moral character, but rather in its consequences. For Kant, if some action implies using someone as a means to an end, then that action is wrong, even if it leads to greater good. That is why killing the potential organ donor is wrong, but killing the person tied to the track is not wrong.

Most legislations follow these Kantian principles, and medical ethics is for the most part deontological. The rule of first doing no harm holds most of the time. Yet, even in those cases where some harm must be done, the Kantian principle still applies: the harm done to someone must never be a means to achieve an end.

As an example consider vaccines. Although pseudoscientists and popular media often exaggerate (to the point of being grossly irresponsible) (14), it is nevertheless true that vaccination campaigns do cause some harms, sometimes even deaths. Vaccinators are responsible for these deaths, yet, by doing so, they are able to save a far greater number of people who would otherwise die of preventable diseases. Strictly speaking, vaccinators face the dilemma of killing a few versus letting many die. If the principle of non-maleficence were to be applied very strictly, then vaccinators should refrain from administering vaccines, because after all, they do cause some harm. Yet, vaccines are considered a great moral good. This is because the case of vaccines is of the same class as the bystander who must pull the lever, and of a different class than the surgeon who thinks of killing a person to distribute his organs to save five patients.

Vaccines are considered a moral good, inasmuch as those few deaths are only side effects, and not the means, to save the greater number of lives. If somehow vaccines could be administered and no deaths would occur as a result, the plan to save more lives would not be shattered. That is how sometimes doing harm may indeed be justified.

***3.      The Third, Fourth and Fifth Trolley Cases***

Apart from the Kantian approach, philosophers have also devised another important concept as a caveat to the primacy of non-maleficence: the doctrine of double effect. Although this doctrine had many antecedents, it was first formally proposed by Thomas Aquinas in the context of military ethics (15). Aquinas is one of the great contributors to the Just War tradition, i.e., a philosophical consideration about how and when it is morally acceptable to wage war.

Aquinas acknowledged that, in every war, innocent lives will be lost, but that need not morally invalidate military actions. Civilians’ deaths are morally acceptable, as long as they come as a result of what in military jargon is called “collateral damage”. Granted, this rather unfortunate phrase has been abused in recent times by reckless politicians and generals, but it still has a legitimate philosophical use.

According to Aquinas, some actions may have not just one effect, but rather two, and the moral quality of those effects may vary. A given action may have one set of good effects, and one set of bad effects. Again, a strict compliance with the principle of non-maleficence would require that those actions never be carried out in the first place, because they will cause some harm, and the prime duty is to first do no harm.

However, Aquinas’ principle of double effect allows for some actions to have bad effects, as long as some conditions are met. First of all, the action itself must be morally good or morally neutral. Second, very much as in Kant’s formulation, the bad effect must not be the means by which the good effect is achieved. Third, the motive must be to achieve only the good effect. And fourth, the good effect must be greater than the bad effect.

In its military applications, this doctrine would allow the bombing of the enemy’s bases, and as a result, the death of some civilians. The bad effects (the civilians’ death) are proportional to the good effects (for example, the destruction of the enemy’s air force), and most importantly, the bad effects are not means to the good effects. If somehow the civilians could survive the bombing, the plan would still hold. This is very different from, for instance, the atomic bombing of Hiroshima. Even if, as sometimes (dubiously) claimed, this atomic bombing brought about the end of World War II, it would still be considered immoral, because the civilians were directly targeted, and their death became the means to the end. If somehow Hiroshima’s civilians survived the atomic bomb, the original plan would not have worked.

The bad effects may be foreseen, but never intended. To get back to medical ethics, consider the case of vaccines previously mentioned. A public health official may foresee that, when a vaccination campaign is begun, some people will die as a result of the vaccines themselves. Yet, the public health official will never intend such deaths, and he will anticipate that the few deaths caused by vaccines are far fewer than the lives saved by the vaccine, thus complying with the requisite of proportionality. The public health official intends to save a greater number of people from dying of preventable diseases; he does not intend to have a very small number of people die from vaccine administration.

Can we then rely on the doctrine of double effect? Thomson herself came up with yet another trolley scenario, in order to test intuitions regarding this doctrine. Consider a trolley that is going on its path, and it is about to run over five people tied to the track. The trolley is about to go underneath a bridge; on that bridge, there is a fat man. If that fat man is pushed over the bridge, his weight will stop the oncoming trolley, he will die, but the five tied to the track will be saved. Should the fat man be pushed?

When asked about the bystander who pulls the lever to redirect the trolley and kill one person, the overwhelming majority of respondents morally authorize the hypothetical bystander. However, when those same respondents are asked about pushing the fat man, the percentage of approval is much lower (11). This is at first strange, because in terms of numbers, both cases are structurally similar: killing one versus letting five die.

However, the doctrine of double effect makes the difference clearer. The bystander foresees the death of the person attached to the track, but does not intend it. By contrast, the person pushing the fat man not only foresees the fat man’s death, but also intends it. The fat man’s death is the means to save the five persons tied to the track.

It may be objected that the fat man’s death is not really intended. Whoever pushed the fat man only wanted him to serve as a buffer against the incoming trolley, and did not wish his death per se. However, proponents of the doctrine of double effect counter that, if in factual terms an action is intrinsically inseparable from its immediate consequence, then that particular consequence must be considered as intended. In that regard, whoever pushes the fat man to stop the trolley, truly intends the fat man’s death, even if that person claims differently.

Again, this has implications on medical ethics and the principle of non-maleficence. Some medical procedures cause harm, but that need not imply that a physician should refrain altogether from administering such procedures. If the action causes harm but also an even greater good, and if the harm is foreseen but not intended, then the action can indeed be carried out.

This principle has applications in two very delicate subjects in medical ethics: abortion and euthanasia. Consider the case of a pregnant woman who has been diagnosed with uterine cancer, and the only way to treat her is by removing the uterus (16). This will end the fetus’ life. Yet, even those religious traditions (especially Catholicism) that are staunchly opposed to abortion, would allow such a procedure, on the basis of the doctrine of double effect. Although the surgeon may foresee that by removing the uterus, the fetus will die, he does not intend it. However, performing an abortion just because the mother’s life is in danger, but directly targeting the fetus, would not be allowed according to Catholic standards. Again, this would not receive moral approval, because the harm would be intended, and not merely foreseen. 

Similarly, consider the case of a terminal patient whose death is imminent and is in severe pain. In order to alleviate pain, the physician administers a dose of morphine, and as a result, the patient dies (17). Is this euthanasia? Not strictly speaking. Although the administration of morphine did cause the death of the patient, it only came about as a result of a morally neutral action, i.e., administering morphine. The doctor may have foreseen, but never intended, the patient’s death. His intention was not to kill the patient, but to relieve his pain. If the patient had survived the morphine shot, the doctor would have been satisfied.

In a case like this, the patient’s condition must indeed be terminal, and her death imminent. After all, death is the greatest harm, and death as a side effect does not seem to be proportional to the action, hence violating the requisite of proportionality. Yet, if the patient is about to die anyways, then the patient’s death may be tolerated as an unintended side effect of the pain alleviating action.

This would be different from, for example, mercy killing. Consider this case, as suggested by Tony Hope: A truck is on fire, with the driver trapped inside. He cannot be saved, and will soon die. The driver has a friend who is outside the tuck with a gun in his hand. If the driver asks this friend to shoot him, he will die a much less painful death than if he burns alive in the flames (18). Hope attempts to make the case that the moral thing to do would be to shoot the driver in order to relieve his desperate pain. Yet, according to conventional medical ethics, relying on the doctrine of double effect, killing the driver would be immoral. Even if the ultimate intention is the relief of pain, there is the mediating intention of killing the driver. As opposed to utilitarianism, the doctrine of double effect does give its share of importance to intentions. In that regard, such a doctrine is part of the deontological understanding of ethics. Harms may be done, but they can never be intended, only foreseen. 

This case of mercy killing suggests that, perhaps in some cases, the doctrine of double effect should be put on hold, given the desperation of the person who asks to be killed. Thomson herself came up with yet another trolley scenario that puts in doubt the relevance of the doctrine of double effect. Consider a trolley that, on its path, will run over five people tied to the track. There is a looping track that eventually goes back to the original track. In that looping track, a fat man is tied. If the trolley is diverted onto the looping track, the weight of the fat man will stop the trolley, and thus, the five lives will be spared. Should a bystander pull the lever to divert the trolley?

Surprisingly, most respondents to this question approve of diverting the trolley in this case (11). This is very strange. In this case, the fat man is used as a means to an end. If somehow he escapes, the plan to save the other five is shattered. The fat man needs to die in order to save the others. His death is not merely foreseen; it is actually intended, as it forms an integral part of the plan. Nevertheless, the same respondents who typically object to throwing down the fat man from the bridge have no qualms about diverting a trolley to run him over, precisely because he is a means to save the five.

This seems to prove that, intuitively, the doctrine of double effect is not as robust as it may appear. In some circumstances, harm can be done, even intentionally so, if it actually leads to a greater good. Thomson put forth this scenario in order to challenge the doctrine of double effect. However, she did not really attempt to offer an explanation as to why the case of the fat man being thrown from the bridge seems morally repugnant, but the case of the fat man being run over by the trolley in the looping track seems to gain moral support. Indeed, it remains mysterious to most philosophers.

Perhaps the difference is that in the case of the fat man being thrown from the bridge, the action is deliberately initiated by the person who throws the fat man. However, in the case of the bystander diverting the trolley in order to run over the fat man, the bystander has not initiated the action, but is only intervening in the last minute. The intervention is clearly direct in the former case, but less so in the latter, and intuitively, this seems to be an important moral difference. In other words, the level of participation in the act seems to be relevant here.

Yet another trolley scenario seems to support this notion. Suppose that the fat man is standing on the bridge, but instead of pushing him off to stop the trolley, someone may pull a lever to open a trap underneath the fat man so that he falls off from the bridge and is run over by the trolley. Should the lever be pulled? Surprisingly, the percentage of respondents who approve such an action is significantly greater than the percentage of respondents who approve of the fat man being thrown by conventional means (19).

This seems to support the intuition that a harm being done, even if both foreseen and intended, is still more excusable if somehow the mechanism is not as direct. This may also have important implications in medical ethics, especially in regard to euthanasia. As of now, most legislations disapprove of euthanasia. In euthanasia, the death of the person is intended, and this is seen as a violation of the moral principle of not intending harm. Yet, if somehow the means to bring about the death of the person were not so direct (such as, for example, assisting the patient’s death instead of directly causing it), then perhaps that could receive greater moral approval. The rule about first doing no harm could be relaxed in favor of a rule allowing harm to be done, as long as the means of doing that harm are not so direct. Thus, the trap door scenario in trolley discussions should be considered in discussions about physician-assisted suicide.

## Conclusion

***Do Intuitions Really Matter?***

A considerable number of variations have been tried in Trolley Problem experiments with surveys, and as a result, psychologists now know better what psychological variables may lead respondents to answer differently. For the most part, it has been established that cognitive overload, reminders of death, and emotional appeals tend to condition subjects to be more deontological in their answers (20). By contrast, exposure to jovial or funny situations tend to condition subjects to be more utilitarian. Similarly, subjects with psychopathic tendencies and brain damage in the ventromedial area are also more likely to take actions to save a greater number of people, disregarding Kantian or double effect principles (21).

Utilitarian philosophers have traditionally given little importance to intuitions. Not surprisingly, most of these philosophers have some affinity with the analytic tradition, and their approach attempts to be entirely cognitive. Cases must be analyzed rationally, regardless of what emotions or intuitions may tell us. This strict reliance on rationality makes utilitarian philosophers much more likely to care about numbers. In their view, the bottom line is, as Bentham famously claimed, to achieve the “greatest good for the greatest number” (22). In all the trolley scenarios, utilitarians would favor whatever option in which the greater numbers of lives are saved. The moral value of an action is not in its intrinsic nature, but rather in its consequences. Utilitarians do not seem to have much patience with the primacy of non-maleficence, precisely because ultimately, this principle may trump achieving the greatest good for the greatest number.

Utilitarians disregard the power and relevance of intuitions. In their estimation, morality should be based on a thorough rational approach, and if that implies overriding some powerful feeling, so be it. Utilitarians legitimately complain that some needed modifications, in both the ethical and legal understanding of medical practice, are stopped because of emotional repulsion rather than rational analysis. In the utilitarian view, there should be no *yuck *factor in medical ethics (23).

Yet, utilitarians may be willing to admit some caveats. Perhaps not in every case should an action be done in order to save more lives. According to most utilitarians, actions should be morally judged on the basis of rules, and not acts (24). A particular action may be considered good in a particular situation, but if it is consistently done in the long term, it may turn out to be very bad. Thus, perhaps throwing the fat man from the bridge would save more lives in that particular moment, but if fat men were consistently thrown from bridges, great panic would prevail in the society, and the consequences could even be worse. As applied to medical ethics, this caveat is relevant. Particular medical procedures should be evaluated for their long-term consequences if they were to become rules.

Whether they judge actions on the basis of actions or rules, utilitarians still believe that this must be done on a rational basis, not an intuitive one. It is undoubtedly true that intuitions can be very deceiving, and a great number of psychological studies have been done to test people on simple cognitive tasks based on intuition, and getting them wrong (25). However, it is still open to debate whether or not moral intuitions can so easily be discarded. After all, as G.E. Moore and other moral non-cognitivist philosophers have long claimed, morality is not about facts (26). In the absence of facts, when it comes to moral judgments, perhaps we ultimately have to rely on intuitions.

Trolley scenarios are not without critics, precisely because some philosophers see no relevance in intuitions when it comes to making moral judgments (27). Other philosophers and psychologists believe the scenarios are too unrealistic to be truly meaningful (28). We may grant that it is extremely unlikely to find five people tied to the tracks of an incoming trolley, and to find a fat man standing on a bridge at that precise moment. Yet, these scenarios are not totally unrealistic, because some structural similarities do remain when we compare them to particular cases of medical ethics as discussed above. For that reason, trolley scenarios will not be the definite criterion in order to decide on things such as euthanasia or abortion, but they may certainly be used as a tool in deliberations on medical ethics.

## Notes:

**Citation to this article:**

Andrade G. Medical ethics and the trolley problem. J Med Ethics Hist. 2019; 12: 3.
